# Protective Effect of *Danshen Zexie* Decoction Against Non-Alcoholic Fatty Liver Disease Through Inhibition of ROS/NLRP3/IL-1β Pathway by Nrf2 Signaling Activation

**DOI:** 10.3389/fphar.2022.877924

**Published:** 2022-06-21

**Authors:** Yaning Biao, Jian Chen, Chenxu Liu, Ruilong Wang, Xue Han, Li Li, Yixin Zhang

**Affiliations:** ^1^ School of Basic Medicine, Hebei University of Chinese Medicine, Shijiazhuang, China; ^2^ International Joint Research Center on Resource Utilization and Quality Evaluation of Traditional Chinese Medicine of Hebei Province, Shijiazhuang, China; ^3^ Hebei Higher Education Institute Applied Technology Research Center on TCM Formula Preparation, Shijiazhuang, China; ^4^ School of Pharmacy, Hebei University of Chinese Medicine, Shijiazhuang, China; ^5^ Shijiazhuang Obstetrics and Gynecology Hospital, Shijiazhuang, China; ^6^ School of Pharmacy, Hebei Medical University, Shijiazhuang, China

**Keywords:** non-alcoholic fatty liver disease, *Danshen zexie* decoction, ROS/NLRP3/IL-1β, Nrf2, oxidative stress, inflammation, pyroptosis

## Abstract

Lipid metabolism disorders are a prominent characteristic in the pathological development of non-alcoholic fatty liver disease (NAFLD). *Danshen zexie* decoction (DZD) is a Chinese herbal medicine that is based on *zexie* decoction and has an effect of regulating lipid mechanism. However, the anti-NAFLD effect and mechanism of DZD remain unclear. In this study, we observed the therapeutic effect of DZD on NAFLD rats and investigated its possible mechanisms. Sixty Sprague Dawley rats were randomly assigned to six groups: control group, model group, *Yishanfu* (polyene phosphatidylcholine) group, and low, medium and high-dose DZD groups. High-fat diet (HFD) was fed to the rats to establish an NAFLD model, and each treatment group was given corresponding drugs at the same time for eight consecutive weeks. The results revealed that the obvious lipid metabolism disorder and liver injury induced by HFD were alleviated by treatment with DZD, which was verified by decreased serum TC, TG, ALT, AST, liver TC, TG, and FFA, as well as the alleviation of hepatic steatosis. The production of ROS in rats was reduced after treatment with DZD. The SOD activity and GSH content were increased with DZD treatment, while the MDA level was decreased. The administration of DZD could decrease serum IL-1β and IL-18 contents. Moreover, DZD upregulated the expressions of Nrf2, HO-1, GCLC, and GCLM, while it suppressed the expressions of NLRP3, caspase-1, GSDMD, and GSDMD-N. In conclusion, the data showed that DZD can reduce lipid accumulation, alleviate oxidative stress and inflammation, and inhibit pyroptosis in NAFLD rats, which might be ascribed to suppression of the ROS/NLRP3/IL-1β signaling pathway by activation of Nrf2. Overall, these results indicated that DZD is expected to be a therapeutic drug for NAFLD.

## Introduction

Non-alcoholic fatty liver disease (NAFLD) is defined by excess fat in liver and has a multidirectional relationship with metabolic syndrome (Li and Alazawi, 2020). Sedentary lifestyles and the consumption of high-calorie diets are spreading worldwide, which has led to greater prevalence of NAFLD in many countries (Perumpail et al., 2017; Estes et al., 2018). NAFLD has a global prevalence of 25%. In Asia, it is now estimated to be 29.6% and is showing a trend of occurring at younger age (Younossi et al., 2016; Li J. et al., 2019). NAFLD can cause liver cirrhosis, liver cancer, and ultimately, death. Moreover, NAFLD is usually associated with abnormal glucose metabolism and a significantly increased risk of cardiovascular and cerebrovascular complications (Lucas et al., 2018). Thus, researchers are seeking to find therapeutic drugs to decrease the incidence of NAFLD. However, lipid-lowering drugs, insulin sensitizers, antioxidants, and other treatments currently available for NAFLD can cause impaired liver function, lipid metabolism disorders, and congestive heart failure. In clinical practice, *Yishanfu* is commonly used to treat NAFLD, whereas some patients are allergic to benzyl alcohol in *Yishanfu*, resulting in gastrointestinal reaction, headache, convulsion and coma (Kubin and Riekki, 2016). Thus, more effective and safer drugs are needed for the treatment of NAFLD.

Inflammation is one of the most important factors in the development of NAFLD, and NOD-like receptor protein 3 (NLRP3) is considered to play a pivotal role in aggravating liver inflammation in NAFLD (Szabo and Petrasek, 2015). Once activated, it induces caspase-1 activation and then accelerates the secretion of inflammatory cytokines, such as interleukin (IL)-1β and IL-18. Subsequently, hepatic Kupffer cells and hepatic stellate cells are activated through inflammatory cytokines, resulting in the recruitment of inflammatory cells and liver fibrosis. Ultimately, this process promotes the progression of simple fatty liver to non-alcoholic steatohepatitis (NASH) (Li et al., 2021). Besides, pyroptosis can also be induced by activation of NLRP3. Pyroptosis is a pro-inflammatory form of cell death and also plays a vital role in NAFLD (Latz et al., 2013; Eguchi et al., 2014).

Oxidative stress is involved in the activation of NLRP3 (Wang K. et al., 2020). Reactive oxygen species (ROS) are a normal metabolic product of redox reactions. Nevertheless, excess high-fat diet (HFD) may trigger mitochondrial dysfunction, leading to the overproduction of ROS, which contributes to oxidative stress. More importantly, excess ROS can activate the NLRP3 in hepatocytes. Studies have revealed that NLRP3-induced inflammatory-cytokine secretion is inhibited when ROS are not produced, which indicates that ROS play a pivotal role in NLRP3 activation (Liu X. et al., 2017).

Nuclear factor erythroid 2-related factor 2 (Nrf2) is a crucial factor for the adjustment of antioxidant mechanisms, and the suppression of its activation has been demonstrated to trigger lipid accumulation in a cellular model of NAFLD (Xu et al., 2021). Furthermore, activation of Nrf2 signaling gives rise to increase the expressions of Heme oxygenase1 (HO-1), Glutamate-cysteine ligase catalytic subunit (GCLC), and Glutamate-cysteine ligase modifier subunit (GCLM). These are proficient in reducing ROS level and alleviating oxidative stress, thus ameliorating NAFLD. Interestingly, increased Nrf2 activity and inhibition of NLRP3 have a tight correlation (Nigro et al., 2017). Therefore, Nrf2 activation could reduce the production of ROS and suppress NLRP3 activation.

Traditional Chinese medicine (TCM) has been used in China for thousands of years and is believed to have valuable efficacy and fewer side effects than conventional medicine. *Danshen zexie* decoction (DZD) was established through the optimization of *zexie* decoction (ZXD), which consists of *zexie* (*Alisma plantago-aquatica* Linn.) and *baizhu* (*Atractylodes macrocephala* Koidz.). Modern studies have shown that both single ZXD and compound decoctions affect cholesterol metabolism and gene expression, resulting in lower lipid levels (Zhao, 2019). In TCM, phlegm and blood stasis are considered the primary pathogenesis of NAFLD. ZXD has a function of promoting water and removing dampness, and *danshen* (*Salvia miltiorrhiza* Bge.) can activate blood circulation, which dissipates stasis. In addition, the water-soluble compounds of *danshen*, such as salvianolic acid A and salvianolic acid B, were found to alleviate inflammation, which could be used to treat NAFLD (Zeng et al., 2015; Ding et al., 2016). The results of analyzing treatments for NAFLD showed that *danshen*, *zexie*, and *baizhu* were frequently among the top 10 (Ding et al., 2019). Consequently, *danshen* was added to ZXD to obtain DZD. However, it is unclear whether DZD can ameliorate NAFLD and what its possible mechanism is. Therefore, this study explores the potential therapeutic mechanism of DZD in HFD-induced NAFLD rats.

## Materials and Methods

### Reagents and Antibodies

Kits for measuring alanine aminotransferase (ALT, #A112-1-1), aspartate aminotransferase (AST, #A113-1-1), total cholesterol (TC, #A111-2-1), triglyceride (TG, #A110-2-1), free fatty acid (FFA, #042-1-1), superoxide dismutase (SOD, #A0001-3), malondialdehyde (MDA, #A0003-2-2), and glutathione (GSH, #A061-2-1) were provided by Nanjing Jiancheng Bioengineering Institute (Nanjing, China). An interleukin-1β kit (IL-1β, #bsk13006) was acquired from Bioss antibodies Co., Ltd. (Shenzhen, China), and an interleukin-18 kit (IL-18, #ERC010.48) was acquired from Neo Biotechnology Science Co., Ltd. (Beijing, China). Antibodies against HO-1 (#GB12104), GCLM (#GB111827), HRP-conjugated Goat Anti-Rabbit IgG (H + L) (#GB23303), and anti-beta actin mouse (#GB12001) were purchased form Wuhan Servicebo Biotechnology Co., Ltd. (Wuhan, China). Antibodies against Nrf2 (#16396-1-AP), GCLC (#12601-1-AP), NLRP3 (#19771-1-AP), caspase-1 (#22915-1-AP), and gasdermin D protein (GSDMD, #20770-1-AP) were obtained from Proteintech Group, Inc. (Wuhan, China). Antibody against GSDMD-N (#sc-376318) was obtained from Santa Cruz Biotechnology Co., Ltd. (Shanghai, China). 2×SYBR Green qPCR Master Mix (Low ROX) and a SweScript RT I First Strand cDNA Synthesis Kit were from Wuhan Servicebo Biotechnology Co., Ltd. (Wuhan, China).

### Preparation of Experimental Drugs

DZD is composed of three Chinese medicinal herbs: *Salvia miltiorrhiza* Bge. (15 g, # 0071873), *Alisma plantago-aquatica* Linn. (30 g, #0093073), and *Atractylodes macrocephala* Koidz. (12 g, #0092373). Chinese herbal medicine prescription granules were acquired from Guangdong Yifang Pharmaceutical Co., Ltd. (Foshan, China). The mixed medicines were dissolved in distilled water at 100°C. Polyene phosphatidylcholine capsules (*Yishanfu*, #ABJD069B) were bought from Beijing Sanofi Pharmaceutical Co., Ltd. (Beijing, China). These capsules are commonly used in the treatment of fatty liver in clinical practice and as a positive control drug in experiments (Guo et al., 2017). In the experiment, distilled water at 100°C was used to dissolve the *Yishanfu*.

### Animal Experimental Procedures

Healthy specific pathogen-free (SPF) male Sprague-Dawley rats weighing 160–180 g were acquired from Vital River Laboratory Animal Technology Co. Ltd. (Beijing, China; Certificate No. SCXK [Beijing] 2016-0006). The animals were housed in a 12-h light-dark cycle and controlled temperature (22 ± 1°C) with free diet and water. All experimental protocols used in this study were approved by the Research Ethical Committee of Hebei University of Chinese Medicine, and the research was conducted in accordance with the Guide for the Care and Use of Laboratory Animals.

After 1 week of acclimatization, all rats were randomized into six groups (*n* = 10 rats per group): control group, model group, *Yishanfu* group, and low, medium, and high-dose DZD groups (L-DZD, M-DZD, and H-DZD). The control group was fed control chow, while the other groups were fed HFD (78.8% basic feed +15% lard +5% sucrose +1% cholesterol +0.2% sodium cholate) to establish NAFLD. The 8-weeks therapy started at the induction of the NAFLD. Drug dosages were calculated based on the literature (Chen, 2011), and the *Yishanfu* group was given *Yishanfu* at 0.144 g/kg/d by gavage. Different doses of DZD were administered to the DZD groups: 1.16 g/kg/d, 2.32 g/kg/d, and 4.64 g/kg/d. The control group and model group were given equal volumes of distilled water. At the end of the experiment, blood and liver samples were rapidly collected for analysis.

### Biochemical Analysis

The activities of ALT and AST in serum and the contents of TC and TG in serum and liver were determined using a semi-automatic biochemical analyzer. The content of FFA in liver was measured by a manual biochemical method. The xanthine oxidase method, thiobarbituric acid method, and spectrophotometric method were used to detect the SOD activity, GSH level and MDA content in liver, respectively. The serum IL-β and IL-18 levels were detected by ELISA. Operations were conducted using the manufacturer’s instructions.

### Hematoxylin and Eosin (H&E) Staining

Liver tissues from each group were fixed with 4% paraformaldehyde for 24 h and then embedded in paraffin for sectioning after dehydration. Paraformaldehyde-fixed paraffin-embedded liver tissues were cut into 5-μm sections. Slides were stained with HE, and all the staining was observed and photographed under a microscope (Nikon Eclipse E100, Tokyo, Japan).

### Oil Red O Staining

Liver tissues from each group were fixed in 4% paraformaldehyde for 24 h and sectioned by a frozen slicer. The sections were stained with oil red O lipid staining working buffer for 45 min after fixing with 5% formaldehyde solution, washing with 60% isopropanol five times, and then washing with distilled water for 2 min. Sections were stained again with hematoxylin for 2 min, washed with distilled water for 5 min, and dried and sealed with glycerine. The lipid droplets were observed and photographed under a microscope. Image Pro Plus 6.0 was used to measure the staining Area (Area) and integrated optical density (IOD) of oil red O, with IOD (sum)/Area (sum) as the final measurement value.

### Immunofluorescence

We restored the frozen slides to room temperature, eliminated obvious liquid, and marked the objective tissue with a liquid blocker pen. We then added spontaneous fluorescence quenching reagent, incubated it for 5 min, and washed it for 10 min with running tap water. We added ROS staining solution to the marked area, and after incubating at 37°C for 30 min, we washed the slide three times with PBS. Then, we incubated the slides with DAPI solution at room temperature for 10 min and washed them three times with PBS. We then threw away some of the liquid and placed a coverslip on top with anti-fade mounting medium. We performed microscopy detection and collected images by fluorescence microscopy (Nikon, Tokyo, Japan).

### Western Blot Analysis

Total proteins in liver tissue samples were extracted using RIPA lysis buffer and quantified with a BCA protein assay kit. Sequentially, the proteins were boiled at 100°C for 10 min, separated by gel electrophoresis, and transferred to PVDF membranes. After blocking in 5% skimmed milk albumin for 1 h, membranes were incubated overnight at 4°C with the following primary antibodies: Nrf2 (1:1,000), HO-1 (1:1,000), NLRP3 (1:1,000), caspase-1 (1:1,000), GSDMD (1:1,000), GSDMD-N (1:1,000), GCLC (1:100,000), and GCLM (1:100,000). We used 1×TBST to wash membranes three times for 10 min. Then, secondary antibody (1:3,000) was used to incubate membranes for 1.5 h. Lastly, immunoreactive bands were visualized by chemiluminescence and quantified by densitometry using Alpha Analysis Software.

### Quantitative Real-Time Polymerase Chain Reaction (qRT-PCR) Analysis

We used Trizol (Servicebo Biotechnology Co., Ltd., Wuhan, China) to extract total RNA from 100 mg of frozen liver tissues. cDNA was generated with a TianScript RT Reagent Kit (Servicebo Biotechnology Co., Ltd., Wuhan, China). Gene expression levels were found by real-time quantitative PCR. The amplification conditions were initial denaturation at 95°C for 15 min and then 40 cycles of 95°C for 10 s and 58°C for 60 s. The PCR data were analyzed by the 2^−△△Ct^ method. The primer sequences used in this study are listed in Table 1.

**TABLE 1:** Primer sequences.

Gene	Forward	Reverse
*Nrf2*	F:TGGAGACGGCCATGACTGATT	R:CACTGTAACTCGGGAATGGAAA
*HO-1*	F:CAGCATGTCCCAGGATTTGTC	R:CCTGACCCTTCTGAAAGTTCCTC
*GCLC*	F:GCATCTAAGTCCCTCTTCTTTCCAG	R:CTGAAGACAGCAGTTGCCCAT
*GCLM*	F:TGTGATGCCACCAGATTTGACT	R:GCTTCAATGTCAGGGATGCTT
*NLRP3*	F:TCTTTGCGGCTATGTACTATCT	R:TTCTAATAGGACCTTCACGT
*caspase-1*	F:TGCCTGGTCTTGTGACTTGGAG	R:TGTCCTGGGAAGAGGTAGAAACG
*GSDMD*	F:CAGGCAGCATCCTTGAGTGTC	R:CCAAGACGTGCTTCACCAACT
*GSDMD-N*	F:GAATTCATGCCATCGGCCTTTGAG	R:GGATCCATCTGACAGGAGACTGAGC
*β-actin*	F:TGCTATGTTGCCCTAGACTTCG	R:GTTGGCATAGAGGTCTTTACGG

### Statistical Analysis

Data are expressed as the means ± standard deviation. The statistical differences of data were analyzed by SPSS 19.0. Normally distributed data was analyzed using one-way ANOVA followed by Tukey’s HSD test, and lowercase letters indicate statistically significant differences (*p*-value < 0.05).

## Results

### DZD Alleviated Liver Injury in NAFLD Rats

**FIGURE 1:**
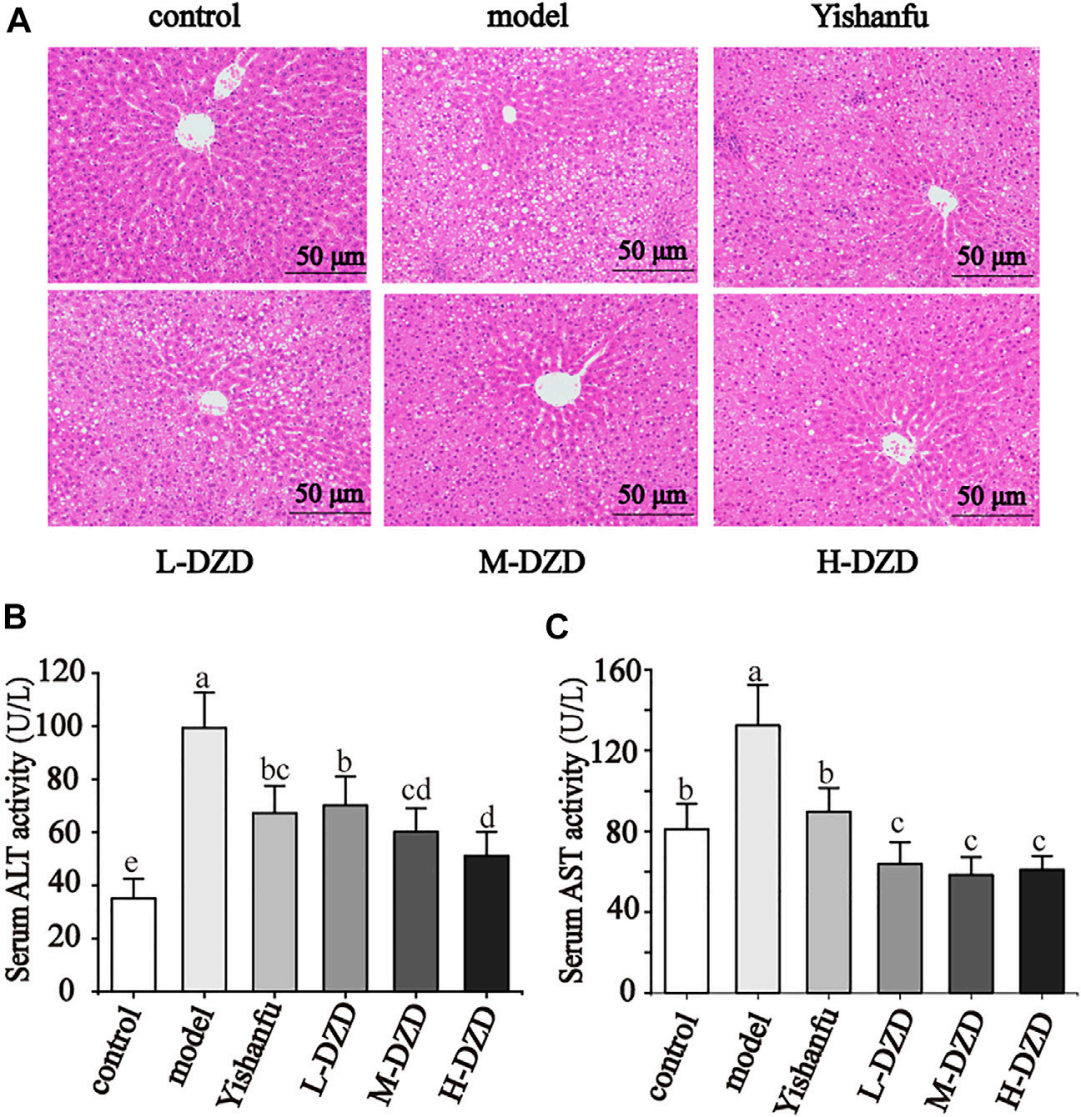
DZD alleviated liver injury in NAFLD rats. **(A)** HE staining of liver tissue (magnification ×200, scale bar = 50 μm). **(B,C)** Serum ALT and AST activities. Data are shown as the mean ± SD, *n* = 10. Lowercase letters indicate statistically significant differences (*p* < 0.05) according to ANOVA followed by Tukey’s HSD test.

NAFLD is always accompanied by liver injury, so we observed liver histopathological changes and the serum ALT and AST activities in rats. As shown in the HE staining result (Figure 1A), liver tissue in the control group showed normal hepatic structure without steatosis. In contrast, a large number of lipid droplet vacuoles and ballooning degeneration appeared in the hepatocytes of the model group. After treatment with *Yishanfu* and DZD, lipid droplet vacuoles and ballooning degeneration were dramatically alleviated, which was more obvious in the H-DZD group.

The results of serum ALT and AST activities are shown in Figures 1B,C. The serum ALT and AST activities in NAFLD rats were obviously higher than the control rats. Rats treated with *Yishanfu* and low, medium, and high doses of DZD showed significant reduction of serum ALT and AST activities. The ALT activity in serum was further decreased in the M-DZD and H-DZD groups compared with the L-DZD group, with the lowest activity in the H-DZD group.

### DZD Regulated Lipid Metabolism in NAFLD Rats

**FIGURE 2:**
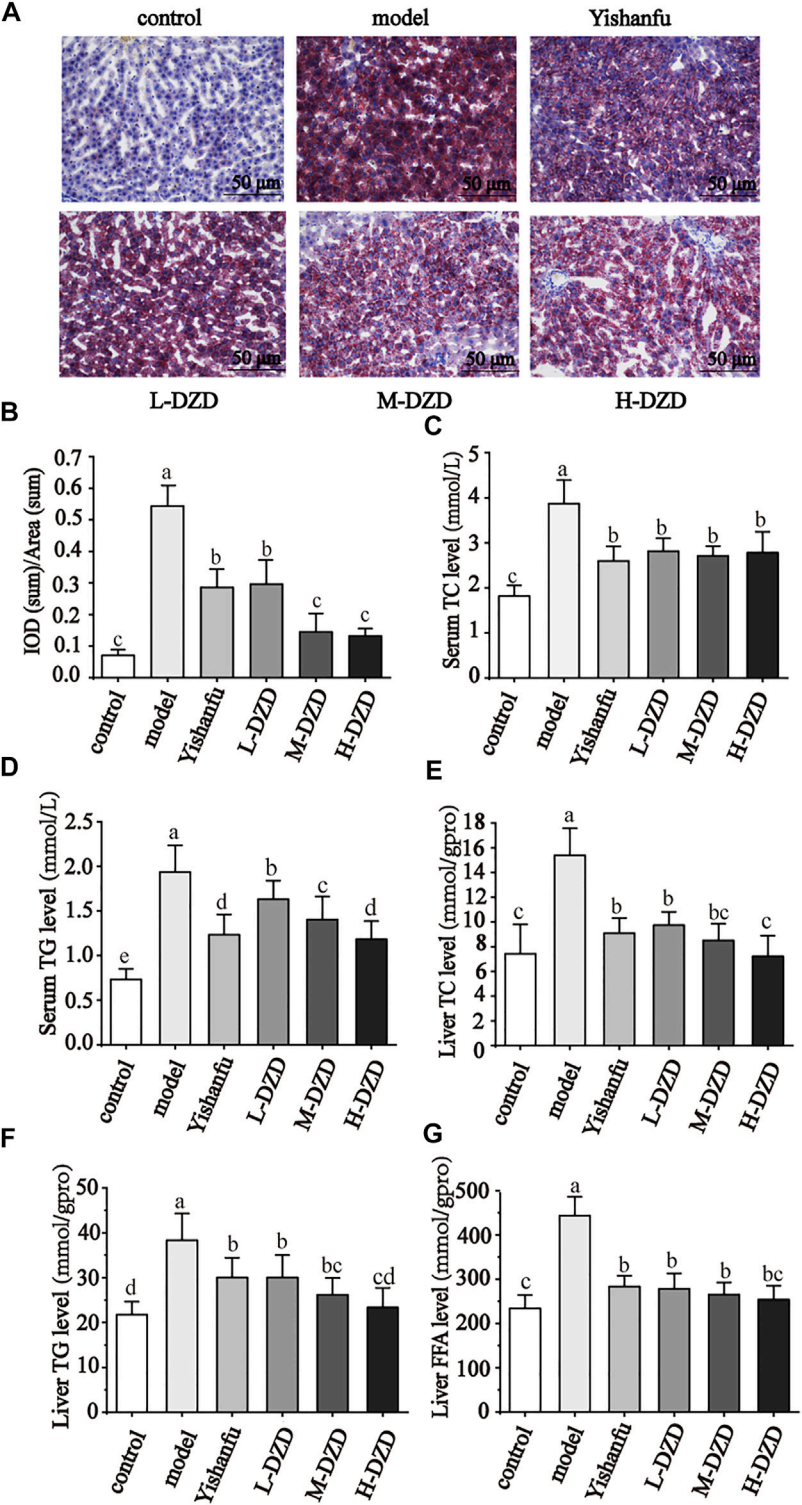
DZD regulated lipid metabolism in NAFLD rats. **(A)** Oil red O staining of liver tissue (magnification ×200, scale bar = 50 μm). **(B)** Quantitative analysis results of oil red O staining. **(C–F)** Serum TC, TG levels and liver TC, TG and FFA levels. Data are shown as the mean ± SD, *n* = 10. Lowercase letters indicate statistically significant differences (*p* < 0.05) according to ANOVA followed by Tukey’s HSD test.

Under normal physiological conditions, the lipid metabolism of the body is in a dynamic and balanced state. Long-term HFD increased the level of FFA, which combines with cholesterol lipid droplets to form TG, causing liver lipid deposition. We used oil red O staining to observe liver steatosis. As shown in Figures 2A,B, a large number of red lipid droplets of different sizes appeared in the hepatocytes of the model rats. Administration of *Yishanfu* and DZD markedly reduced lipid droplets accumulation. Furthermore, the lipid droplets accumulation in the M-DZD and H-DZD groups indicated significant reduction than the *Yishanfu* and L-DZD groups.

In Figures 2C–G, compared to the control group, the serum levels of TC and TG and liver contents of TC, TG, and FFA dramatically increased in the model group. *Yishanfu* and DZD with different doses significantly decreased these levels in rats. Among them, the H-DZD group reduced the serum level of TG and liver contents of TC and TG were most significantly.

### DZD Suppressed Inflammation and Pyroptosis by Inhibiting NLRP3 in NAFLD Rats

**FIGURE 3:**
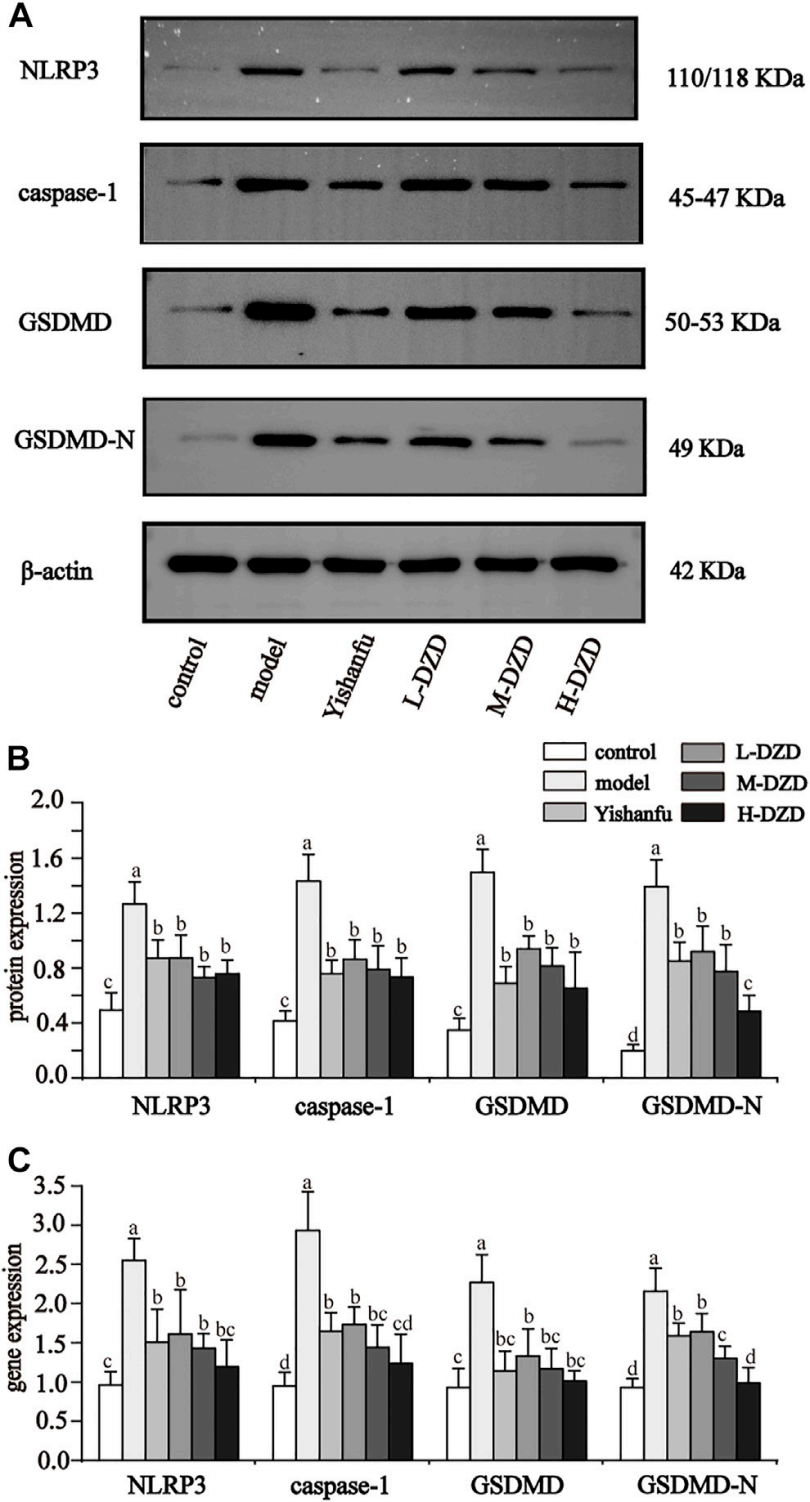
DZD inhibited NLRP3 activation and pyroptosis in NAFLD rats. **(A,B)** Western blot analysis of NLRP3, caspase1, GSDMD and GSDMD-N. Data are shown as the mean ± SD, *n* = 3. **(C)** qRT-PCR analysis of NLRP3, caspase1, GSDMD and GSDMD-N. Data are shown as the mean ± SD, *n* = 6. Lowercase letters indicate statistically significant differences (*p* < 0.05) according to ANOVA followed by Tukey’s HSD test.

**FIGURE 4:**
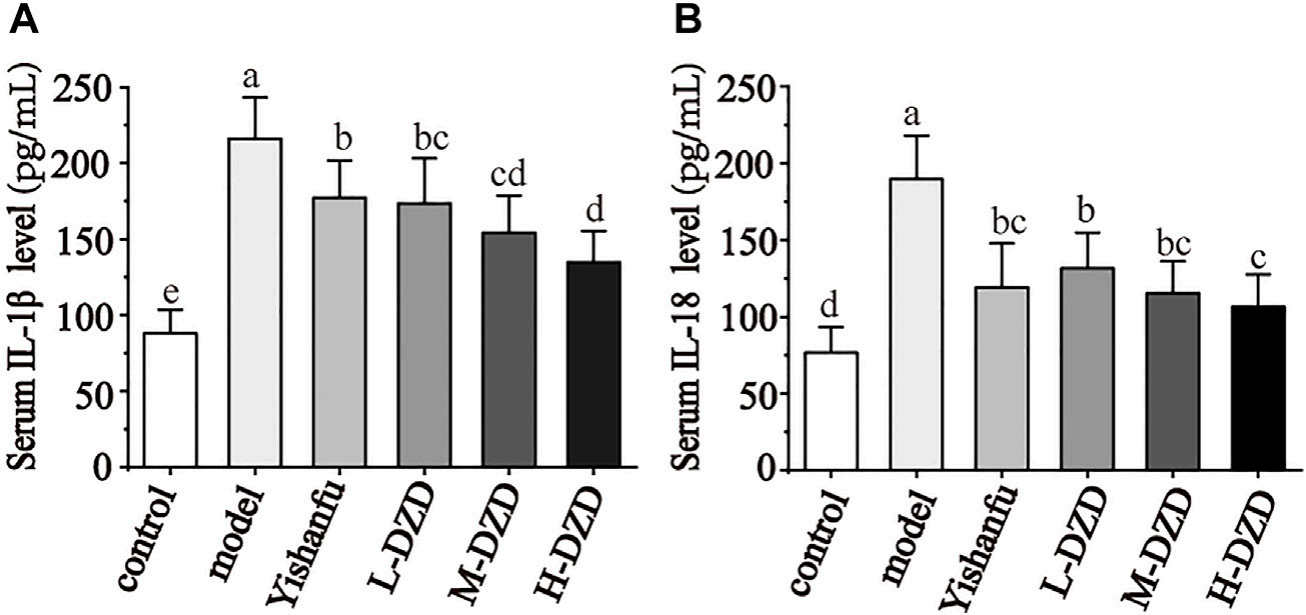
DZD suppressed inflammatory response in NAFLD rats. **(A,B)** Serum IL-1β and IL-18 levels. Data are shown as the mean ± SD, *n* = 10. Lowercase letters indicate statistically significant differences (*p* < 0.05) according to ANOVA followed by Tukey’s HSD test.

NLRP3 activates caspase-1 and promotes the secretion of IL-1β and IL-18 while also triggering GSDMD-driven pyroptosis, which both exacerbate the progression of NAFLD (Zhang et al., 2015; An et al., 2019; Kelley et al., 2019). We investigated the effects of DZD on NLRP3. As shown in Figures 3A–C, the expressions of NLRP3, caspase-1, GSDMD, and GSDMD-N in the model group were significantly increased than the control group. Compared with the model group, the expressions of NLRP3, caspase-1, GSDMD, and GSDMD-N were decreased by *Yishanfu* and DZD. In particular, the gene and protein expressions of GSDMD-N in the H-DZD group were notably decreased than the L-DZD and M-DZD groups. To further explore the anti-inflammatory effect of DZD, we detected serum IL-1β and IL-18 contents by ELISA. As shown in Figure 4, *Yishanfu* and DZD administration markedly decreased serum contents of IL-1β and IL-18.

### DZD Relieved Oxidative Damage in NAFLD Rats

**FIGURE 5:**
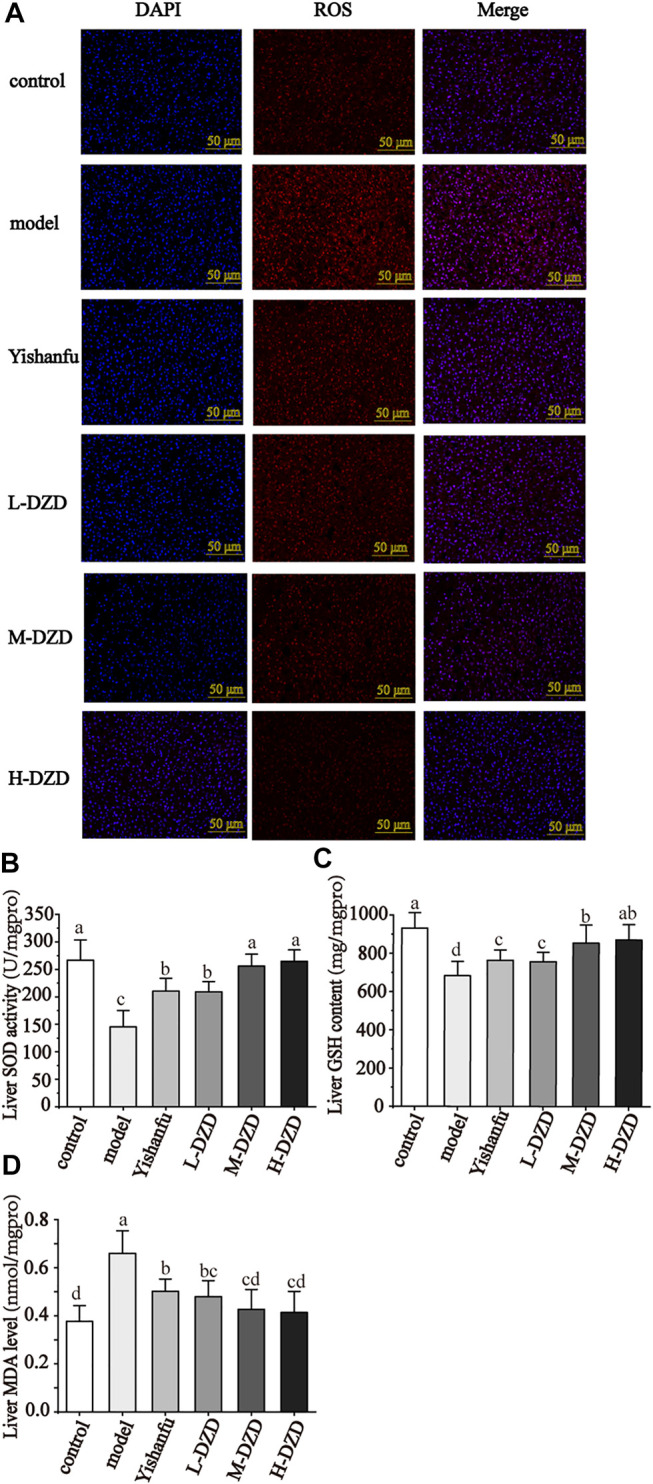
DZD relieved oxidative damage in NAFLD rats. **(A)** ROS production in liver by immunofluorescence (magnification ×200, scale bar = 50 μm). **(B–D)** Liver SOD activity, GSH content and MDA level. Data are shown as the mean ± SD, *n* = 10. Lowercase letters indicate statistically significant differences (*p* < 0.05) according to ANOVA followed by Tukey’s HSD test.

Next, ROS production and oxidative stress biomarkers were detected. As shown in Figures 5A–D, in the model group, the SOD activity and GSH content in liver were dramatically decreased, and the liver levels of ROS and MDA were markedly increased. *Yishanfu* and DZD significantly increased the SOD activity and GSH content and reduced the ROS and MDA levels. In addition, both the M-DZD and H-DZD groups drastically increased the SOD activity and GSH content than the L-DZD group.

### DZD Activated Nrf2 Signaling in NAFLD Rats

**FIGURE 6:**
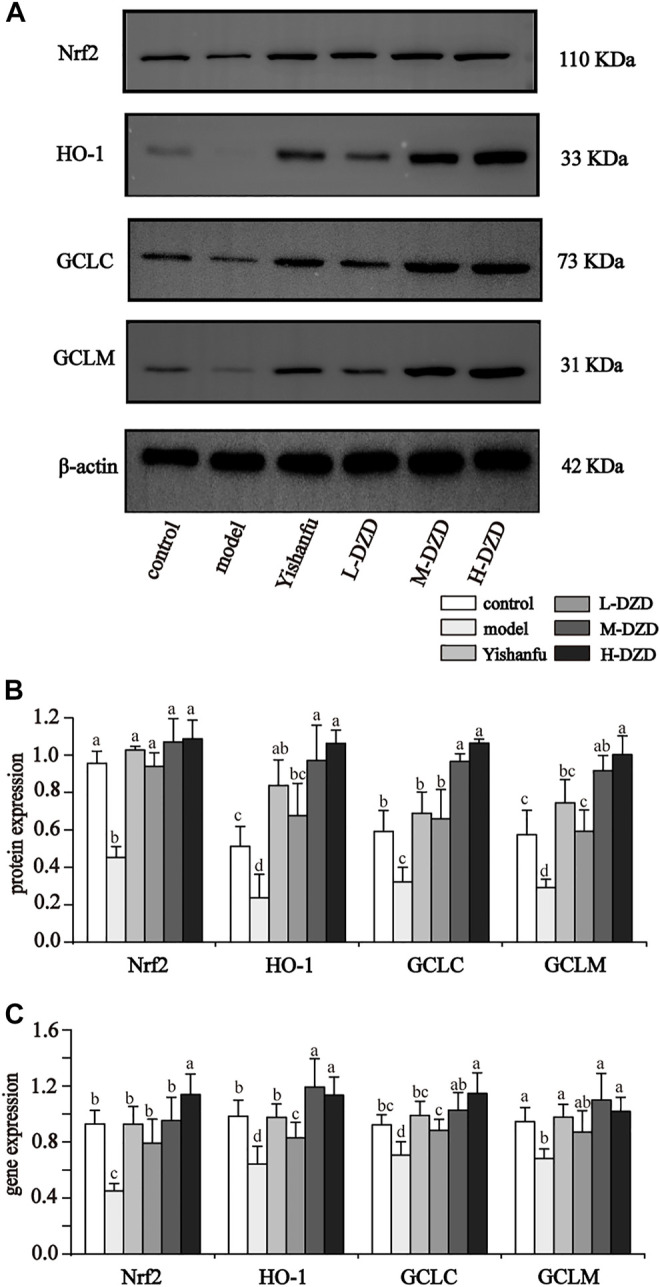
DZD activated Nrf2 signaling in NAFLD rats. **(A,B)** Western blot analysis of Nrf2, HO-1, GCLC and GCLM. Data are shown as the mean ± SD, *n* = 3. **(C)** qRT-PCR analysis of Nrf2, HO-1, GCLC and GCLM. Data are shown as the mean ± SD, *n* = 6. Lowercase letters indicate statistically significant differences (*p* < 0.05) according to ANOVA followed by Tukey’s HSD test.

Oxidative stress is regarded as a catalyst of NLRP3 activation. To investigate the role of Nrf2 in inhibiting NLRP3 in NAFLD rats, we measured the expressions of Nrf2, HO-1, GCLC, and GCLM by western blot and qRT-PCR. As shown in Figures 6A–C, the administration of *Yishanfu* and DZD upregulated the expressions of Nrf2, HO-1, GCLC, and GCLM. Compared to the L-DZD group, the protein expressions of HO-1, GCLC and GCLM were obviously increased in the M-DZD and H-DZD groups, with the most significant effect seen in the H-DZD group. The expressions of HO-1 mRNA and GCLC mRNA were markedly increased in the M-DZD and H-DZD groups than the L-DZD group. Meanwhile, the H-DZD group increased the expression of Nrf2 mRNA was the most significant.

## Discussion

NAFLD is characterized by discomfort in the right hypochondriac region, increased body weight, abdominal distention, fatigue, and loss of appetite. According to the pathogenesis and clinical characteristics of NAFLD, TCM pertains to categories of “hypochondriac pain,” “phlegm turbidity,” “accumulation,” and so on. TCM theory indicates that the liver stores blood and regulates the smooth flow of “*qi*,” while the spleen governs transportation and transformation of water and dampness, as well as promotion of *qi* and blood. The occurrence of NAFLD is considered to be mostly due to irregular diet, emotional disorder, or sedentary lifestyle, resulting in spleen dysfunction in transportation and liver malfunction in dispersion. Due to this process, phlegm and blood stasis are accumulated in liver.

NAFLD is mainly related to the liver and spleen as phlegm-blood stasis is key to its pathogenesis. ZXD is derived from the “*Synopsis of the Golden Chamber*,” and DZD’s component characteristics correspond to the pathogenesis of NAFLD. *Zexie* has a remarkable effect of removing dampness, while *baizhu* could strengthen the spleen, remove dampness, and promote diuresis, and *danshen* has the effect of promoting blood circulation and removing blood stasis. Thus, DZD may effectively eliminate phlegm, strengthen the spleen, facilitate blood circulation, and remove blood stasis.

Currently, the pathogenesis of NAFLD is complicated, and the “two-hit” theory proposed by Day and James in 1998 has been universally received to describe the underlying pathogenic mechanism (Anurag et al., 2017). Abnormal lipid metabolism is the catalyst in the pathological course of NAFLD. In the “first hit” of the theory, insulin resistance induces liver steatosis, which triggers lipid metabolism disorders and liver damage (Brea and Puzo, 2013). As seen in the results, in the model group, the lipids contents increased significantly in serum and liver. All treatment groups showed a reduction of the serum levels of TC, TG, and liver contents of TC, TG, and FFA. Moreover, combined with the results of the ALT and AST activities, it was shown that DZD could regulate lipid metabolism and alleviate liver injury. However, there was no significant difference in serum AST activity among each dose group of DZD. The probable cause is that AST activity is not as specific as ALT for liver injury. The increased AST activity also reflects the extent of myocardial damage, which is a distraction to the result (Fan et al., 2020).

Subsequently, the second hit occurs on the basis of the first hit. Lipid storage (from the first hit) induces a series of cytotoxic events in the hepatocytes. The second hit is oxidative stress, which is triggered by increased production of ROS (Simon et al., 2019). Lipid peroxidation continues to lead to aggravated fatty degeneration of the liver, leading to its deterioration (Takaki et al., 2013). Eventually, inflammation occurs in liver and gradually develops into NASH.

In normal liver tissue, oxidation and antioxidant systems are in a state of dynamic balance, and physiological ROS can be effectively removed through intracellular antioxidant mechanisms. Nevertheless, in the case of NAFLD pathology, excessive FFA and lipid accumulation in liver introduce lipotoxicity, leading to mitochondrial dysfunction and the production of ROS, which exceeds the body’s antioxidant capacity (Ogawa et al., 2018). The growing production of ROS and the decreased activity of the ROS-scavenging mechanism trigger the overaccumulation of ROS, giving rise to oxidative stress damage and the generation of lipid peroxides, such as MDA.

Furthermore, the excessive ROS production can activate NLRP3-mediated inflammation, strengthening the generation of inflammatory cytokines and ultimately promoting the progression of simple fatty liver to NASH (Machado and Diehl, 2016; Lee J. et al., 2019; Lee Y. et al., 2019). When ROS production is absent, NLRP3 gives rise to impaired production of IL-1β, which reveals the fundamental role of ROS in the activation of NLRP3 (Hewinson et al., 2008). In this research, DZD administration markedly decreased ROS production and alleviated oxidant stress.

The NLRP3 inflammasome is a multiprotein complex that consists of NLRP3 associated with the adapter protein, apoptosis-associated speck-like protein, and pro-caspase-1 (He et al., 2017). It has been verified that NLRP3 is an important part of the development of NAFLD, and ROS produced by oxidative stress can activate the NLRP3 inflammasome, leading pro-caspase-1 to be converted to activated caspase-1. Next, activated caspase-1 converts the inactive pro-IL-1β and pro-IL-18 into mature IL-1β and IL-18 (Tourkochristou et al., 2019). The increased content of IL-1β could upregulate fatty acid synthase and promote lipid accumulation in hepatocytes (Negrin et al., 2014). IL-18 could activate nuclear factor-κB and further induce inflammation (Neumann et al., 2002). Ultimately, IL-1β and IL-18 could be secreted from the cells and induce a cascade of inflammation. In our present research, DZD decreased serum inflammatory factor contents of IL-1β and IL-18 as well as the expressions of NLRP3 and caspase-1 in liver, indicating DZD could suppress liver inflammation.

Moreover, activated caspase-1 cleaves GSDMD into a N-terminal fragment and a C-terminal fragment. GSDMD-N is a main cause of pyroptosis and GSDMD-mediated pyroptosis plays a critical role in NAFLD (Wang et al., 2021). Various cells in liver participate in pyroptosis. Macrophages are the most important innate immune cells involved in pyroptosis and are crucial to the damage process implicated in steatohepatitis (Shi et al., 2015; Liu Y. G. et al., 2017). Recent study has also identified hepatocyte pyroptosis as an important contributor to liver injury and fibrosis (Gaul et al., 2021). GSDMD-N has the ability to move to the cell membrane and generate pore-forming activity, releasing large amounts of pro-inflammatory factors that accelerate the development of NAFLD (Wang L. et al., 2020). In human NAFLD/NASH liver tissues, GSDMD is increased compared to normal conditions (Xu et al., 2018). In addition, GSDMD-N levels are related to the NAFLD activity score (Beier and Banales, 2018). It was reported that inhibition of NLRP3 could significantly improve liver function and blood lipids in NAFLD rats (Oh et al., 2021). In this present study, DZD significantly decreased the gene and protein expressions of GSDMD, and GSDMD-N in liver.

Nrf2 is a member of the CNC-bZIP family member and a fundamental molecule in the antioxidant mechanism. It can trigger the expressions of anti-oxidative genes to combat oxidative damage. Research has shown that Nrf2 is a potential target in the mitigation of NAFLD (Gao et al., 2021). Under control circumstances, Nrf2 binds to Kelch-like ECH-associated protein1 (Keap1) and exists in the cytoplasm in an inactive state. When activated by a stimulus, Nrf2 dissociates from Keap1 and translocates to the nucleus, combining with ARE and then activating downstream anti-oxidative gene transcription. This contributes to decreasing the ROS content and restraining oxidative stress (Aleksunes and Manautou, 2007; Bataille and Manautou, 2012; Yang et al., 2018). HO-1, GCLC, and GCLM are all downstream genes of Nrf2 (Iida et al., 2004). HO-1 can alleviate oxidative damage (Li Z. et al., 2019), GSH has the ability to scavenge free radicals, and GLC is a rate-limiting enzyme for the synthesis of GSH and is made up of GCLC and GCLM. If the expression of both is reduced, it can promote the synthesis of GSH in the body, thereby accelerating the elimination rate of ROS (Higuchi et al., 2007). SOD is mainly produced by hepatocytes and could scavenge excessive ROS in the body, as well as inhibit lipid peroxidation (Nguyen et al., 2020). A previous study demonstrated that activation of the Keap1/Nrf2 pathway reduced lipid accumulation in liver of mice fed HFD (Shin et al., 2009). This finding is consistent with previous reports showing that HFD may reduce the expression of Nrf2 in liver (Soto-Alarcon et al., 2019; Ortiz et al., 2020), while DZD increased the expressions of Nrf2 and its downstream genes. DZD also reversed the SOD activity and GSH content, and decreased the excessive ROS and MDA. However, the mechanism used by DZD to activate Nrf2 has not been clarified, which needs to be studied further.

**FIGURE 7:**
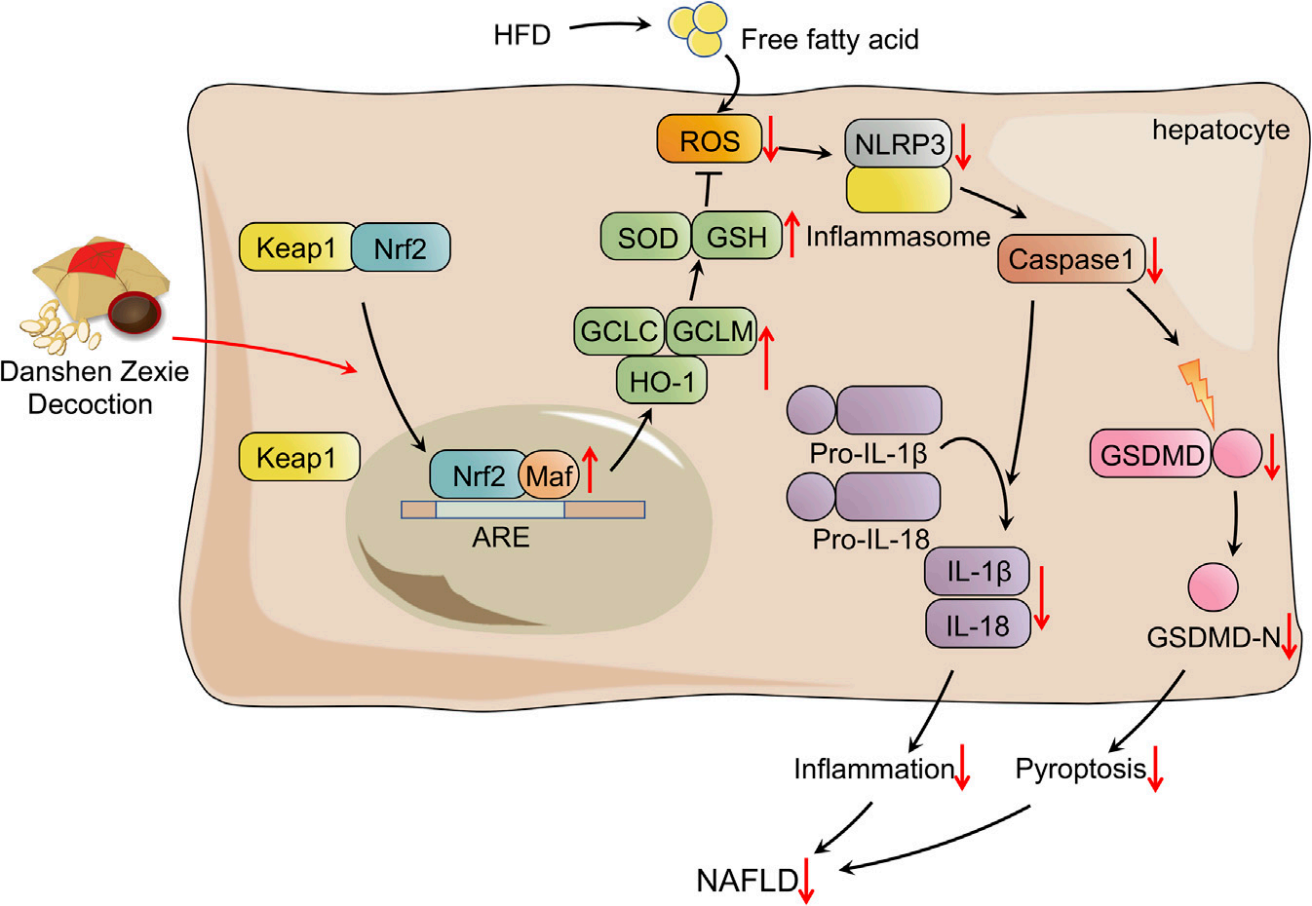
Schematic presentation probable treatment mechanism of DZD on HFD-induced NAFLD.

In conclusion, the overall findings of the present study illustrated that DZD protects against HFD-induced NAFLD by suppressing the ROS/NLRP/IL-1β signaling pathway by activating Nrf2, which inhibits oxidative stress, inflammation, and subsequent pyroptosis (Figure 7). The research has provided sufficient evidence that DZD has potential as a therapy for NAFLD. Nonetheless, further research *in vivo* and *in vitro* will be needed to make it DZD applicable for protecting against NAFLD.

## Data Availability

The original contributions presented in the study are included in the article/Supplementary Material, further inquiries can be directed to the corresponding authors.
